# Dual mode fluorescence and spectrophotometric cefixime sensing using onion juice nitrogen doped carbon dots with smartphone and paper strip readouts

**DOI:** 10.1038/s41598-025-31426-y

**Published:** 2025-12-12

**Authors:** Shahin Asadi, Negar Ziraksaz

**Affiliations:** UNAM — National Nanotechnology Research Center and Institute of Materials Science and Nanotechnology, Bilkent University, Ankara, Turkey

**Keywords:** Smartphone imaging, Eco-friendly carbon dots, Ratiometric absorbance, Cefixime analysis, Smartphone and test-strip readouts, N-doped carbon dots, Synergistic quenching, Chemistry, Environmental sciences, Optics and photonics

## Abstract

The widespread presence of cefixime (CFX) in the environment has raised concerns about potential risks to ecosystems and human health, including bronchitis, tonsillitis, and laryngitis. Therefore, developing an accurate and convenient on-site analysis device for CFX is crucial. Herein, a dual-mode fluorescence–spectrophotometric platform with smartphone and test-strip readouts was designed to detect CFX. A microwave method was demonstrated for synthesizing N-doped fluorescent Carbon dots (N-CDs) by using onion juice as a precursor, with optimization achieved through Box-Behnken Design (BBD). XPS, HRTEM, XRD, FTIR, TRF, UV-Vis spectrophotometry, and fluorescence were used to investigate the N-CDs structure and optical properties. N-CDs displayed satisfactory optical properties, an extraordinary quantum yield of 72.4%, an average particle size of 6.12 ± 0.5 nm, excellent water solubility, high ionic strength, and outstanding pH stability. The limits of detection (LOD) for the fluorescence, spectrophotometric, smartphone, and test strip were 5.16 nM, 138.08 nM, 0.56 µM, and 0.65 µM, respectively. Detection operates via a synergistic quenching mechanism (inner-filter effect plus dynamic quenching). In real samples (honey and tap water), recoveries of 94.21–108.83% with RSD ≤ 1.87% confirmed the accuracy of quantification. This developed platform provides a reliable, environmentally friendly, and efficient platform for on-site CFX monitoring in environmental applications.

**Supplementary Information:**

The online version contains supplementary material available at 10.1038/s41598-025-31426-y.

## Introduction

 Cefixime (CFX) is a semi-synthetic, third-generation cephalosporin antibiotic. It is active against both Gram-positive and Gram-negative bacteria, as well as microbial infections such as bronchitis, tonsillitis, laryngitis, gonorrhea, and pneumonia. CFX treats urinary tract infections, upper respiratory tract infections, skin infections, and middle ear infections^1,2^. Antibiotic residues, such as CFX, can linger in water and enter food chains; even trace residues contribute to driving antimicrobial resistance and pose cumulative risks to consumers. Because centralized testing is costly and slow for complex matrices, there’s a clear need for sensitive and rapid portable assays. This study has shown that nanomaterial-based fluorescence and spectrophotometric platforms, combined with smartphone or paper-based readouts, enable fast and reliable screening of food and environmental samples^3,4^. Several techniques, such as spectrophotometry^5,6^, chromatography^7–9^, capillary electrophoresis^10^, spectrofluorimetric^11–13^, and electrochemical methods^14,15^, have been reported for monitoring CFX in biological or pharmaceutical samples.

Carbon dots (CDs) are nanoparticles with sizes below 10 nm whose abundant defects and surface states yield bright, tunable photoluminescence and enable facile surface functionalization. They have attracted tremendous attention for favorable properties such as high quantum yield^16^, low toxicity^17^, good biocompatibility^18^, excellent fluorescence stability^19^, broad and continuous excitation^20^, and strong resistance to photobleaching^21^.In comparison with heavy-metal quantum dots or noble-metal clusters^22,23^, CDs are metal-free, and generally more biocompatible; many other nanoparticles, they are synthesized by simple, often green routes and pair naturally with paper and smartphone-based readouts for on-site testing^24,25^. These advantages motivate our use of N-doped onion juice-derived CDs as the signal transducer for CFX sensing. CDs have been applied in various fields, including analytical chemistry^26^, bioimaging^27^, and drug release^28^, among others.

Green-synthesized, biomass-derived nanoparticles, especially CDs, offer sustainable transducers for fluorescence and spectrophotometric sensing. They are inexpensive to produce at scale, avoid toxic precursors, and provide strong optical readouts. These traits translate to portable assays that couple naturally with paper strip/smartphone formats for on-site analysis of food and environmental samples^29–31^.

Heteroatom doping is a promising strategy to improve the optical properties of CDs and increase the quantum yield (QY)^32^. In particular, the nitrogen (N) atom can combine strongly with the C atom due to its similar size to C; therefore, nitrogen-doped CDs (N-CDs) often exhibit higher fluorescence QYs than undoped CDs^33,34^. Various routes have been reported for synthesizing CDs, including hydrothermal methods^35^, laser ablation^36^, electrochemical oxidation^37^, and microwave methods^38^. Among these, Microwave-assisted synthesis is a low-cost and straightforward method, affording short reaction times and often reducing the need for harsh conditions compared to conventional thermal routes^39^.

Fluorescence-based methods have attracted much attention due to their outstanding features, such as great sensitivity, simplicity, fast responses, relatively low cost^40,41^, monitoring analyte-induced changes in emission intensity, enabling very low LODs, and good tolerance to matrix effects^42,43^. Compared with instrument-intensive, high-cost laboratory methods, fluorescence platforms offer rapid screening with strong quantitative reliability, making them well-suited for food and environmental monitoring, as well as smartphone and paper-based readouts^44,45^.

Recently, the smartphone and test strip readout, a portable and easy-to-operate device, has been used extensively due to the detection platform’s good sensitivity and specificity, simplicity, and visualization, which have made it an attractive tool for the practical detection of analytes^46,47^ in comparison with costly methods. This approach is low-cost and field-ready^48^. These sensing platforms can employ a self-checking method by comparing the individual mode detection outcomes and are validated using fluorescence and spectrophotometric methods to achieve more reliable and accurate results^49^.

**Fig. 1:**
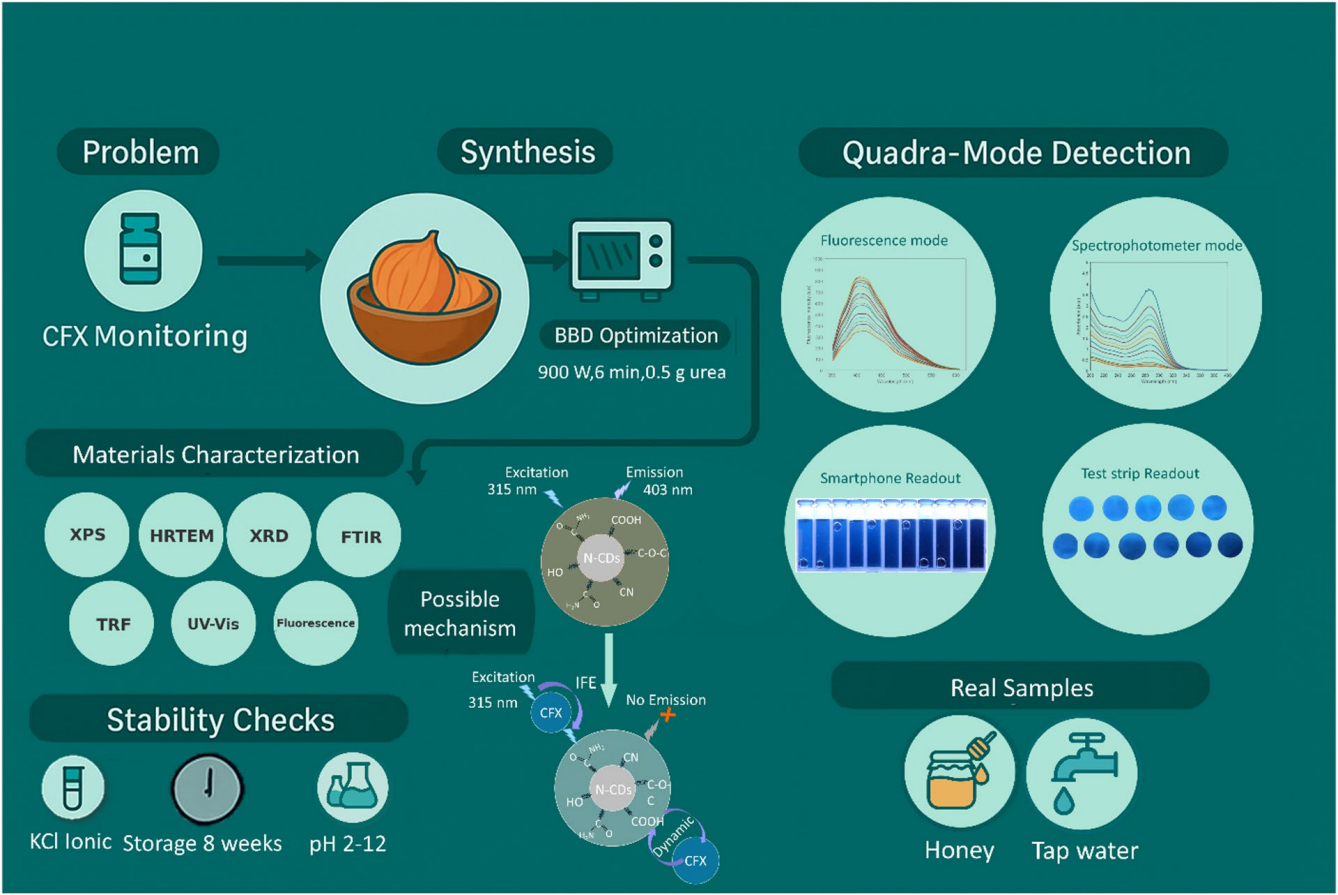
Schematic illustration of the dual mode fluorescence and spectrophotometric cefixime sensing platform using onion juice nitrogen doped carbon dots with smartphone and paper strip readouts.

Prior CFX assays (Table 2) show relatively high LODs and single-mode readouts^50–53^. To overcome these disadvantages, we developed a dual-mode platform with smartphone and paper-based readouts for CFX detection. N-CDs were prepared from onion juice and urea via a one-step microwave route. The synthesis conditions were optimized through Box–Behnken design (BBD), resulting in a remarkable quantum yield of 72.4%. The N-CDs morphology, composition, surface functionalities, and particle size were subsequently characterized. The sensor had a wide linear range with satisfactory detection limits. To our knowledge, no prior study has combined both optical methods with both portable readouts for CFX using green N-CDs. An overview of the dual-mode cefixime sensing strategy with dual readouts is shown in Fig. 1.

## Experimental procedures

### Materials and reagents

Onion was collected from a local vegetable market in Tehran, Iran, in January. Unless stated, chemicals were analytical grade (Sigma-Aldrich). Cefixime (CFX, ≥ 98%); quinine sulfate dihydrate (QY reference; in 0.10 M H₂SO₄); urea (≥ 99.5%, Merck Ltd.); Na₂HPO₄ (≥ 99%) and NaH₂PO₄ (≥ 99%) for 0.10 M phosphate buffer (pH = 6.0); NaOH (≥ 98%); citric acid (≥ 99.5%); L-cysteine (≥ 98%); glucose (≥ 99%); ascorbic acid (≥ 99%). Salts for ionic-strength/interference tests: KCl (≥ 99.5%), MgCl₂ (≥ 98%), CaCl₂ (≥ 96%), MnCl₂ (≥ 98%), ZnCl₂ (≥ 98%), TiOSO₄·xH₂O (analytical grade), Na₂SO₄ (≥ 99%). Selectivity probes: cefazolin sodium, cefotaxime sodium, amoxicillin trihydrate, aspirin (acetylsalicylic acid), atenolol, metformin hydrochloride, clopidogrel bisulfate, vancomycin HCl, and azithromycin dihydrate (all Sigma-Aldrich). 0.22 μm syringe filters (Membrane Solutions), and ultrapure water (18.2 MΩ·cm) were used throughout.

### Instrumentation

The synthesized N-CDs from onion with urea were validated through various analytical instruments. The X-ray photoelectron spectroscopy (XPS) evaluated the elemental composition of N-CDs, which was analyzed by Thermo Scientific K-Alpha XPS. A high-resolution transmission electron microscope (HR-TEM) was used to study the surface morphology of the N-CDs, captured using a Tecnai G2 F30 (300 kV). Fourier transform infrared spectroscopy (FTIR) was used to study the surface functionalities of the N-CDs and was recorded using a PerkinElmer Spectrum Two. The spectrum was recorded over the range of 4000–500 cm^− 1^ with an average scan rate of 30 scans. Powder X-ray diffraction (XRD) patterns of the N-CDs were collected on a JEOL JDX-3532. A PerkinElmer LS45 fluorescence spectrophotometer was used to carry out the fluorescence measurements. Time-resolved fluorescence (TRF) lifetimes were measured on a HORIBA FluoroLog spectrofluorometer. UV–Vis spectrophotometer (LAMBDA 1050) was used to analyze the UV–Vis absorption spectra of N-CDs.

### Synthesis of N-CDs

Several onions were selected, their outer layers were removed, and they were washed with deionized water. The cleaned onions were then cut into small pieces. Subsequently, the onion pieces were processed using a Philips juicer to obtain onion juice. The extracted juice was transferred to Falcon tubes and centrifuged. In the first filtration step, the sample was centrifuged at 6000 rpm for 10 min, and the supernatant was collected. For further purification, the supernatant was subjected to a second centrifugation at 15,000 rpm for 15 min. The obtained supernatant was then filtered through Whatman No. 42 filter paper, and the filtrate solution was stored at 4 °C for further synthesis experiments.

The N-CDs were synthesized using a one-pot household microwave method. A total of 20 ml of filtered onion juice and 0.5 g of urea were mixed in a 250 mL beaker and sonicated for 5 min. The mixture was then transferred to the microwave and irradiated at 900 W for 6 min with periodic cooling every 2 min. The resulting dried residue was sonicated for 5 min with 20 mL of distilled water, then centrifuged at 15,000 rpm for 20 min to remove large particles. Subsequently, the solution was filtered through a 0.22 μm cellulose nitrate syringe filter membrane to remove the impurities and unreacted raw materials. The filtrate was then dialyzed (MWCO 3.5 kDa) against ultrapure water (18.2 MΩ·cm) for 24 h at room temperature, with five water changes. Finally, the clear black-brown colored supernatant liquid was then transferred to the oven at 55°c for 96 h to obtain a black solid and stored at 4 °C for an additional to experiments.

### Design of the experiment

A standard Box-Behnken design approach was applied to optimize the synthesis of N-CDs. three independent variables, microwave power (X1), irradiation time (X2), and the gram weight of urea (X3), were selected as the variables. Fluorescence was examined as a dependent response. The experimental design was implemented using Design-Expert version 11. Based on preliminary experiments, all variables were fixed at three levels: X1 (500, 700, 900 W), X2 (3, 6, 9 min), and X3 (0.1, 0.3, 0.5)^54^. The design of experiments was conducted to maximize the quantum yield by optimizing the primary synthesis variables. A total of 17 runs were performed, comprising 12 distinct runs and 5 center points. The synthesized carbon dots were diluted at a fixed ratio, and their fluorescence responses were measured; the input data used in the software are listed in **Table ****S1**. After entering these inputs, we conducted the statistical analysis shown in **Table S2**: the model is significant (p-value < 0.05) with an F-value of 525.37. All parameters are significant except the interaction between irradiation time and urea amount, and the lack of fit is not significant. We then performed a qualitative assessment. As seen in **Fig. ****S1**, the diagnostic plots indicate a good fit and model agreement. **Fig. S2** displays the 3D response surfaces and contour plots, which confirm the quadratic fit and show the two-factor interactions.

### Determination of the quantum yields

The fluorescence quantum yield (QY) of the prepared N-CDs was measured as per the following Eq. (1):

1 $${\mathrm{QY}}_{{\mathrm{S}}} = {\text{ QY}}_{{{\mathrm{ST}}}} \left[ {{\mathrm{M}}_{{\mathrm{S}}} /{\mathrm{M}}_{{{\mathrm{ST}}}} } \right]\left( {\eta ^{{\mathrm{2}}} _{{\mathrm{S}}} /\eta ^{{\mathrm{2}}} _{{{\mathrm{ST}}}} } \right)$$

QY_S_ and QY_ST_ are the quantum yields of the N-CDs solution and standard, respectively. Quinine sulfate (In 0.1 M H2SO4, with QY = 0.54) was used as the standard for these measurements. M_S_ and M_ST_ are the slopes of the linear plot between the integrated intensity of fluorescence and the UV–Vis absorbance of the N-CDs and standard, respectively. Ƞ is the refractive index of the solvents used to dissolve the N-CDs and the standard^55^.

### Detection procedures (fluorescence and spectrophotometric) with smartphone and test-strip readout

In this section, we describe the preparation procedures for two modes (fluorescence and spectrophotometric) and two readouts (smartphone and test strip). First, because fluorescence, spectrophotometric, and smartphone use similar solution preparation procedures with minor adjustments, we address them together. For solution preparation, identical 10 mL volumetric flasks from the same brand are arranged in a row, and 1 mL of the N-CDs solution is pipetted into each flask (27.5 µg mL⁻¹ for fluorescence, 110 µg mL⁻¹ for spectrophotometric and smartphone measurements). Several flasks are reserved as blanks, and 1 mL of CFX solution at different concentrations is added to the remaining flasks to cover the calibration range. Finally, the solutions are brought to volume with PBS buffer at pH 6, previously identified as the optimal working pH for CFX^56^, and filled to the mark. They are then incubated for 2 min to ensure homogeneity and transferred to 1 cm cuvettes for measurements.

For the fluorescence mode, the cuvette is placed in the spectrofluorimeter. At an excitation wavelength of 315 nm, the emission spectra of the N-CDs with and without CFX are recorded over 300–800 nm at a scan rate of 250 nm min⁻¹. The emission peak at 403 nm is then used for subsequent analyses to construct the calibration curve and to evaluate the relationship between drug concentration and emission intensity.

For the spectrophotometric mode, the UV-Vis spectrophotometer is first baseline-corrected with the blank, then the cuvette is inserted. Absorbance spectra of the N-CDs, in the presence and absence of CFX, are recorded from 200 to 800 nm. The relationship between CFX concentration and absorbance change is quantified by ratiometric calibration using the ratio of A_287_/A_247_ (where A_287_ and A_247_ represent the absorbance at 287 and 247 nm, respectively). This self-referencing normalization, which divides the analyte-responsive band by a proximal reference wavelength, mitigates errors arising from lamp-intensity fluctuations, baseline drift, and matrix-induced turbidity, thereby improving calibration robustness.

For the smartphone readout, the cuvette is placed at the center of a light-shielded black box (30 × 15 × 20 cm) to minimize disturbing light. A smartphone (Samsung S22 Ultra) is positioned 14 cm from the cuvette at a 60° angle relative to a 315 nm UV excitation source, matching the fluorescence excitation wavelength. Camera settings (focus, ISO, exposure, and white balance) are locked. Images are acquired for N-CDs with and without CFX, and the relationship between the extracted color values and CFX concentration is used to construct the calibration curve.

For the test strip readout, Whatman Grade 1 chromatography paper is punched into 6 mm disks. The disks are first soaked in an N-CDs solution (300 µg mL⁻¹) for 5 min and then vacuum-dried at 40 °C for 10 min to obtain N-CDs test strips. The test strips are then soaked in CFX solutions at varying concentrations for 10 min and subsequently vacuum-dried at 40 °C for 10 min to prepare the strips^57^. Because the test strips are intended for portable use and to bring laboratory capability to the point of need, 365 nm illumination was employed in this study, which is widely available and low-cost. As smartphone cameras are sensitive to UV light and may introduce noise, this can help ensure that imaging is feasible with any phone. All elements, including the box and the angular settings of the camera, light source, and smartphone, are the same as in the smartphone readout. After imaging, the relationship between the extracted color codes and CFX concentration is used to construct the calibration curve.

### Actual sample

Considering the practicality of CFX, the two real samples were estimated using the N-CDs fluorescent probe. The tap water was directly obtained from the laboratory and centrifuged at 15,000 rpm for 20 min. A honey sample was collected from the natural hive. 5mL of honey was mixed with 5 mL of PBS buffer (pH 6) using a vortex mixer for 5 min, and then centrifuged at 4000 rpm for 10 min. Finally, the supernatant was diluted to 100 mL^58^.

## Results and discussion

### Characterization

**Fig. 2:**
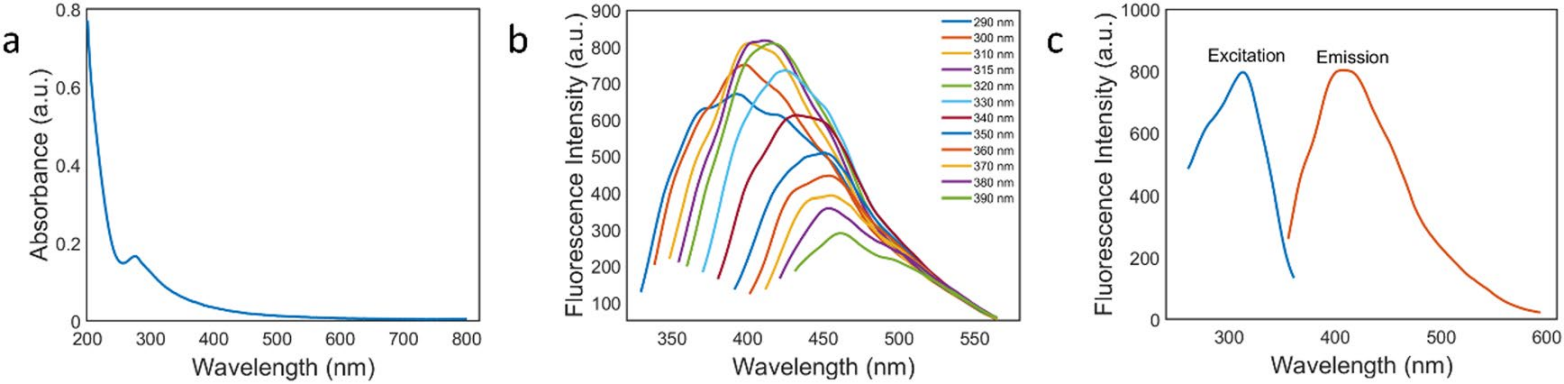
**a** UV–Vis absorbance spectrum, **b** Excitation dependent spectra, **c** Excitation and emission spectra of N-CDs.

The UV–Vis spectrum of the N-CDs displays a single broad near-UV absorption band with an intensity maximum around ~ 287 nm (Fig. 2a). This band is dominated by π→π transitions of aromatic sp² domains with contributions from n→π* transitions of surface carbonyl/imine groups (C = O/C = N). In many CDs, the short-wavelength π→π* feature merges with or is not visible due to strong background absorption, which is consistent with the literature on N-doped/biogenic CDs^59^.

Using Eq. (1), the fluorescence quantum yield of the N-CDs was QY = 0.724 (72.4%). This high QY is consistent with literature showing that nitrogen incorporation and surface passivation in carbon dots enhance PLQY by enriching radiative states and suppressing non-radiative traps^55,60^. The fluorescence characteristics were investigated at various excitation wavelengths ranging from 290 to 390 nm in 5 nm intervals. As shown in Fig. 2b, the emission peaks increased between 290 and 315 nm, after which the intensities decreased, and the peaks exhibited a slight red shift. Upon excitation at 315 nm, N-CDs showed the maximum fluorescence emission intensity at 403 nm. The excitation and emission spectra were recorded to examine the fluorescence behavior, as shown in Fig. 2c.

**Fig. 3:**
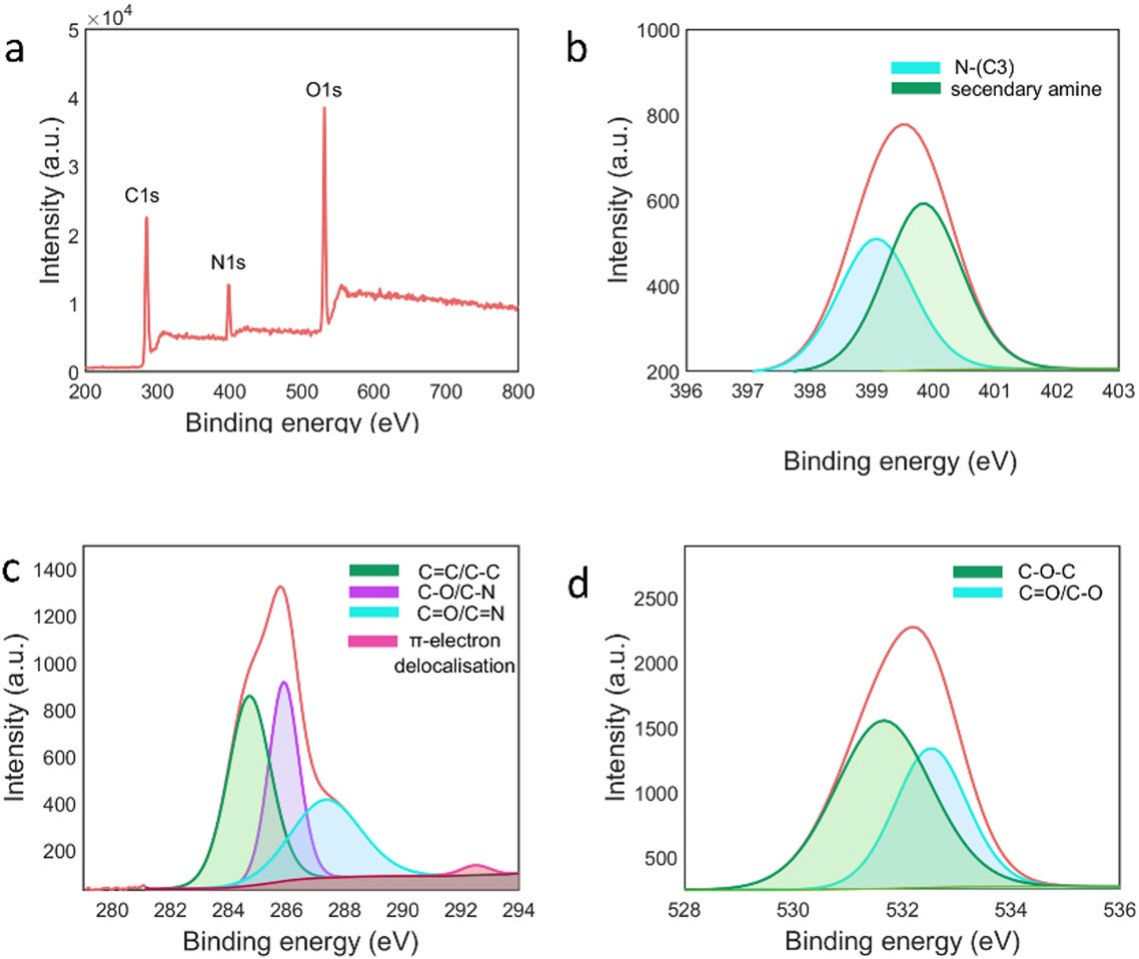
**a** Survey graph, High-resolution spectra of, **b** N1s, **c** C1s, **d** O1s of N-CDs.

An XPS analysis determined the N-CDs surface composition and oxidation states. The survey spectrum in Fig. 3a shows peaks at 284.75, 399.53, and 531.13 eV, corresponding to N (11.78%), C (57.37%), and O (30.85%), respectively. N1s spectra Fig. 3b showed peaks at 399.08 and 399.88 eV corresponding to N-(C_3_) and secondary amine, respectively^61^.C1s Fig. 3c high-resolution spectra showed four peaks at 284.68, 285.88, 287.38, and 292.48 eV ascribed to C − C/C = C, C − O/C − N, C = O/C = N, and π-electron delocalisation groups^61–63^, respectively. O1s spectra exhibited peaks at 531.68 and 532.58 eV, corresponding to C− O− C and C = O/C− O, respectively (Fig. 3d)^61,63^. The occurrence of these functional groups indicates the hydrophilicity on the N-CDs surface, which aligns with FTIR results.

**Fig. 4:**
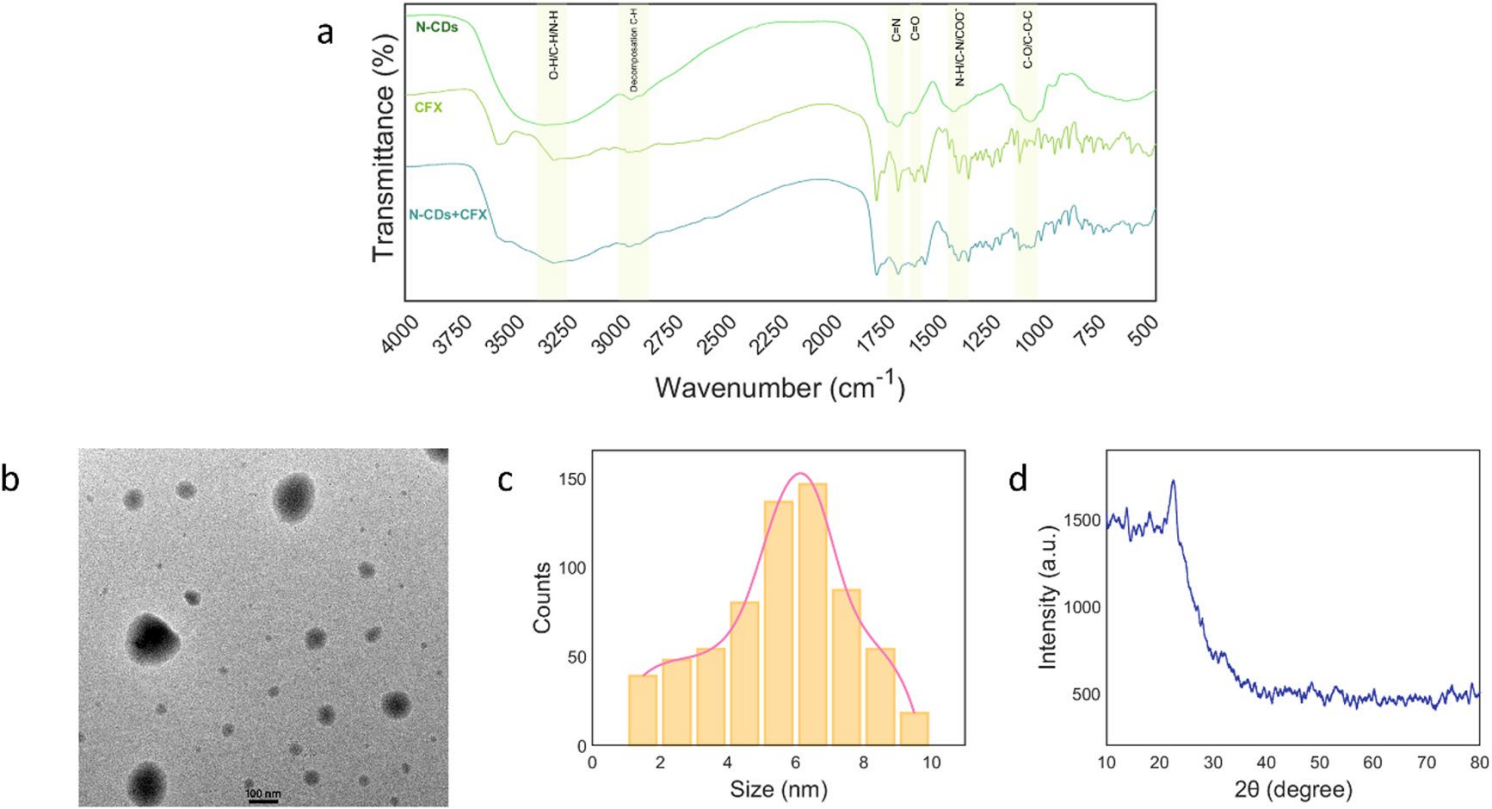
**a** FT-IR spectra of onion juice-derived N-CDs, CFX, and N-CDs + CFX. **b** High resolution transmission electron microscopy (HR-TEM) image )scale bar = 100 nm), **c** Histograms chart, **d** XRD spectrum of N-CDs.

The FTIR spectra in Fig. 4a show a broad peak at 3100–3400 cm^− 1^, corresponding to the O − H/N − H/C-H stretching vibrations. The band appears at 2936 cm^− 1^, which signifies C − H decomposition. and signal at 1714 cm^− 1^ are attributed to C = N stretching vibrations^64^ and signals at 1676 and 1408 cm^− 1^, respectively attributed to C = O and N − H/C − N/COO^-^^65,66^. Signals at 1044 cm^− 1^ resemble C − O/C − O − C stretching^63^.

FT-IR spectra of N-CDs after interaction with CFX showed significant changes, indicating a chemical interaction. A broad band at 3336 cm⁻¹ suggests hydrogen bonding from N–H or O–H groups. The C = O stretching peak at 1676 cm⁻¹ broadened, indicating involvement of the carbonyl group. The 1408 cm⁻¹ band also broadened, likely due to aromatic or C–N shifts. A sharper peak at 1044 cm⁻¹ indicates structural rearrangement or new bonds in the C–O region. These shifts confirm successful conjugation of CFX to N-CDs through multiple functional groups.

Figure 4b illustrates the High-Resolution TEM image (HR-TEM) of N-CDs, which reveals quasi-spherical, uniform, and well-dispersed nanoparticles. The histogram in Fig. 4c displays the particle size distribution, yielding an average particle size of 6.12 ± 0.5 nm, with diameters ranging from 1.5 to 9.5 nm^67^.

The X-ray diffraction (XRD) analysis presented in Fig. 4d reveals a single broad peak at 2θ = 22.6°. This peak is associated with the (002) planes of turbostratic graphitic carbon, exhibiting a d-spacing of around 0.393 nm. The width of the peak suggests the existence of small, disordered graphitic domains within the N-CDs^68^.

### Stability studies

**Fig. 5:**
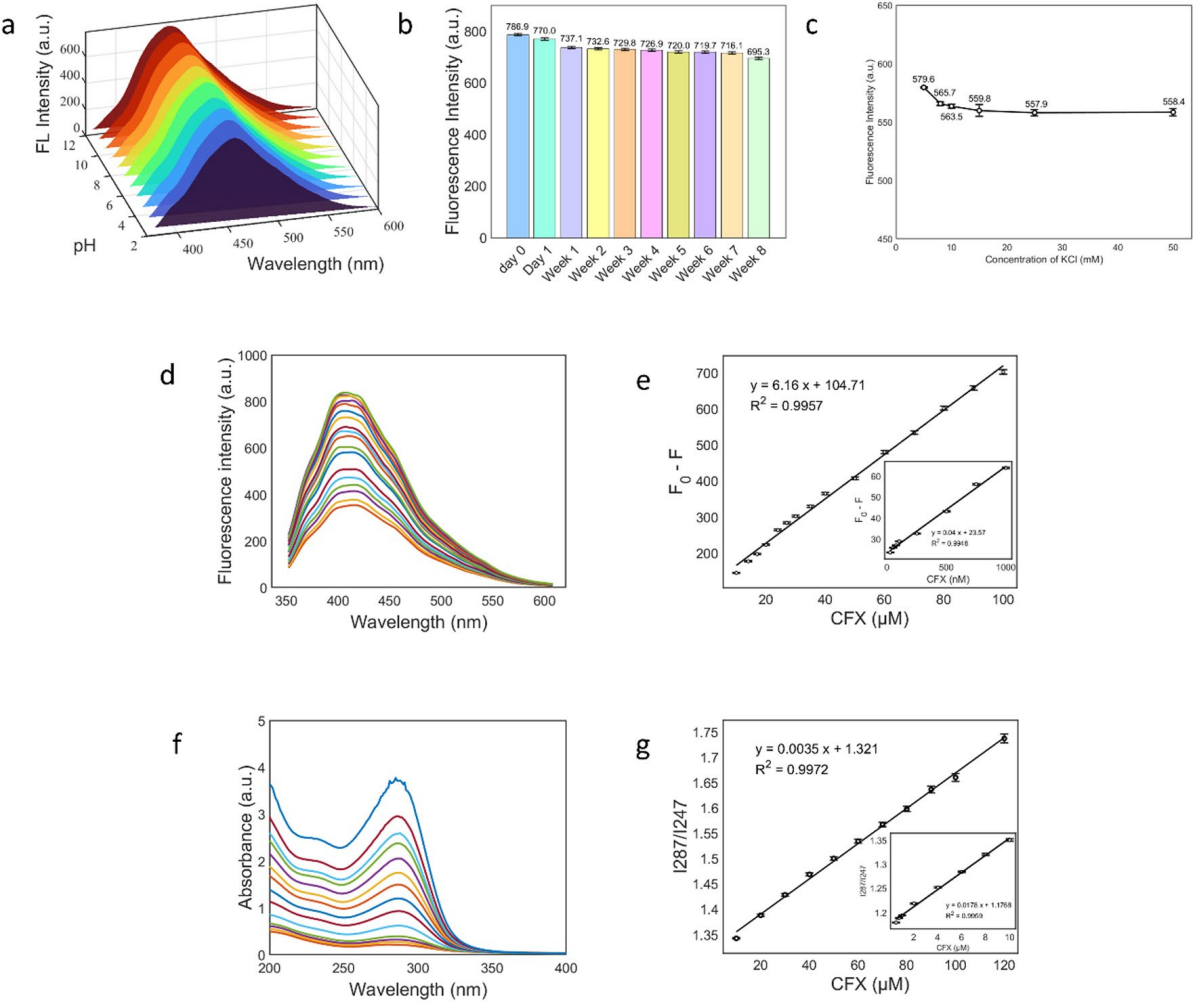
**a** Variation of fluorescence intensity with pH, **b** Variation of fluorescence intensity with storage time, **c** Variation of fluorescence intensity with ionic strength of N-CDs. d Fluorescence emission spectra of N-CDs with increasing CFX concentrations, showing concentration-dependent quenching. **e** Calibration of the fluorescence response (F₀-F) vs. CFX concentrations; linear ranges: 10–100 µM (main) and 25–1000 nM (inset). **f** Spectrophotometric spectra of the N-CDs/CFX system with increasing CFX concentrations. **g** Calibration of the absorbance (A₂₈₇/A₂₄₇) vs. CFX concentrations; linear ranges: 10–120 µM (main) and 0.5–10 µM (inset).

The stability of the N-CDs was assessed in relation to pH levels ranging from 2 to 12, storage duration of 8 weeks at 4 °C, and ionic strength with KCl concentrations ranging from 5 to 50 mM under optimized optical conditions. As shown in Fig. 5a, the fluorescence intensity increases from pH 2 to 4, then increases gradually from pH 5 to 10, reaching a maximum near pH 11, before slightly decreasing at pH 12. This pH dependence aligns with the protonation and deprotonation of surface emissive states, which affects the radiative recombination in the N-CDs^69,70^.

Long-term storage tests demonstrated high emission retention, as shown in Fig. 5b. The signal decreased from 786.9 a.u. on day 0 to 695.3 a.u. after 8 weeks, reflecting an approximate loss of 11.7% over 56 days, which averages to about 0.21% per day. Notably, there was no detectable spectral shift during this period, indicating a good shelf-life.

The fluorescence stability of the as-prepared N-CDs was investigated under varying ionic strengths. The fluorescence intensity of the N-CDs remained nearly unchanged across different concentrations of KCl, as shown in Fig. 5c. This demonstrates that the system is stable even at higher ionic strengths, making it suitable for application in various ionic conditions^71^.

### Analytical performance for CFX detection: fluorescence & spectrophotometric; smartphone/test-strip

Using two analytical modes (fluorescence and spectrophotometric) and two readouts (smartphone and paper test strip), we established the calibration relationship between signal and CFX concentration for detection.

Fluorescence method: As previously described, the solutions were prepared, and their fluorescence emission spectra were recorded using an excitation wavelength of 315 nm. The maximum emission intensity of the N-CDs alone was denoted as F₀. In contrast, the maximum emission intensities in the presence of various concentrations of CFX were denoted as F. Figure 5d shows a progressive decrease in fluorescence intensity with increasing CFX concentration, attributed to a concentration-dependent quenching effect. Plotting F₀ – F versus CFX concentration revealed two distinct linear regions with strong correlation coefficients, indicating high sensitivity and a consistent quenching response over a broad concentration range (Fig. 5e**)**.

Spectrophotometric mode: Following the preparation of the solutions and spectrophotometer setup, UV-Vis absorption spectra were recorded for both pure N-CDs and N-CDs in the presence of varying concentrations of CFX. Analysis focused on the absorbance ratio between the peak at 287 nm and the trough at 247 nm (A_287_/A_247_). As illustrated in Fig. 5f, an increase in CFX concentration resulted in a noticeable rise in absorbance and a progressive increase in the A_287_/A_247_ ratio. This trend enabled the construction of two linear calibration curves correlating absorbance ratio with CFX concentration, confirming the reliability of this ratiometric approach (Fig. 5g**)**.

**Fig. 6:**
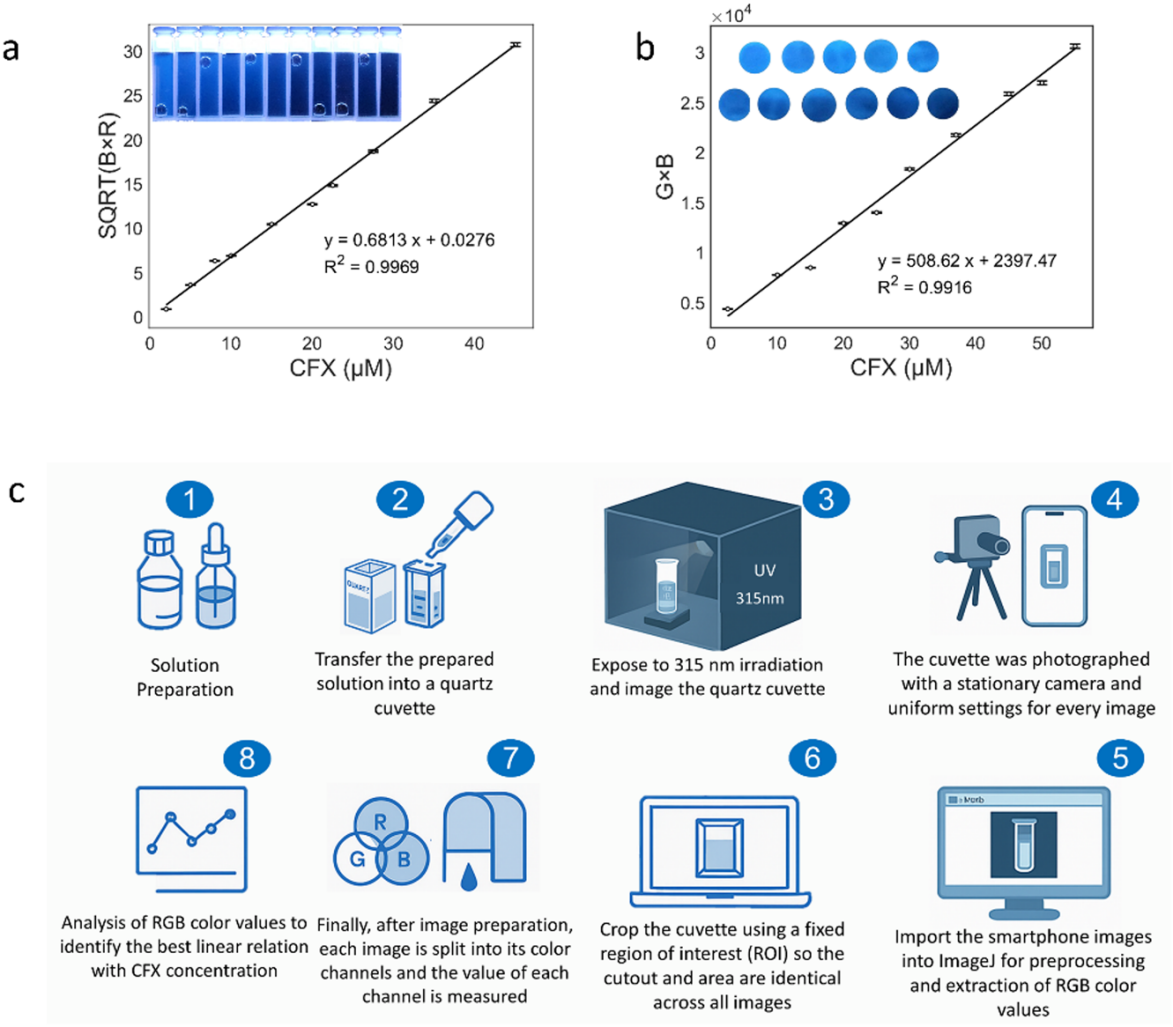
**a** Smartphone calibration: response √(B×R) vs. CFX concentrations (2–45 µM); inset: smartphone photos of N-CDs/CFX solutions. **b** Paper test-strip calibration response: (B × G) vs. CFX concentrations under 365 nm UV (2.5–55 µM); inset: strips at increasing concentrations. **c** Smartphone imaging schematic; the test strip uses the same capture and image-processing method.

Image acquisition and processing (smartphone and test-strip readouts). After imaging under the defined conditions, photographs were transferred to a laptop and processed in ImageJ (NIH). For each format, a region of interest (ROI) was specified: for the smartphone fluorescence readout, only the inner cuvette area was selected; for the test-strip readout, only the circular test disks were selected. Images were then split into the R, G, and B channels, and the mean pixel intensity within the ROI was extracted for each image containing N-CDs in the presence and absence of CFX. The extracted channel intensities were tabulated alongside CFX concentration, and the linear relation with the highest coefficient of determination (R²) was used for quantitation. Accordingly, for the smartphone readout, the best linear fit was given by √(B×G), which relates the blue and green intensities to CFX concentration (Fig. 6a), whereas for the test-strip readout, the product (B×G) performed best (Fig. 6b). The difference reflects the distinct optical properties and illumination geometry of the cuvette versus the paper substrate. These choices provided the best linearity and noise performance, acting as self-calibrated readouts that mitigate illumination/geometry effects. A schematic overview of this workflow is shown in Fig. 6c.

In dual-mode fluorescence–spectrophotometric sensing with smartphone and test-strip readouts, the detection limit was calculated using 3σ/K, and the limit of quantification using 10σ/K, where σ is the standard deviation and K is the slope of the linear line. LOD and LOQ values for all methods are detailed in Table 1.

Table 2 summarizes recent studies using carbon dot-based fluorescent sensors for CFX detection. Dual-mode fluorescence–spectrophotometric sensing with smartphone and test-strip readout shows superior sensitivity, a wider dynamic range, lower LOD and LOQ, and greater applicability in real sample analysis, showcasing its potential as an efficient platform for CFX detection.

**Table 1:** Sensing capabilities for CFX by the prepared sensor.

Parameters	Fluorescence	Spectrophotometric	Smartphone	Test strip
Range	10–100 µM 25-1000 nM	10–120 µM 0.5–10 µM	2–45 µM	2.5–55 µM
Limit of detection	34.35 nM 5.16 nM	0.76 µM 138.08 nM	0.56 µM	0.65 µM
Limit of quantification	114.50 nM 17.20 nM	2.54 µM 460.11 nM	1.88 µM	2.19 µM
Regression equation	R^2^ = 0.9957 R^2^ = 0.9946	R^2^ = 0.9972 R^2^ = 0.9959	R^2^ = 0.9969	R^2^ = 0.9916

**Table 2:** Different quantum dot-based sensing devices for CFX detection.

Sr. No.	Sensor system	Linearity range	Detection limit (LOD)	Real sample tested	Refs
1	CDs nanosensor	0.2-8 µM	0.05 µM	human urine	^50^
2	MIP-CdS QDs	0.001–0.7 mg mL^-1^	0.54 ng mL^-1^	pharmaceutical and urine samples	^51^
3	BS-CQDs	250nM to 15 µM	169 nM	Tablet sample and Mimic biological environment	^52^
4	B, N-CD nanoparticles	0–100 nM	4.21 nM	honey, tap water, seawater	^53^
5	N-CDs	from low nM to µm	5.16 nM (Fluor.) /0.138 µM (Spectrophotometric) / 0.56 µM (Smartphone) / 0.65 µM (Test Strip)	Tap Water and Honey	Current Work

### Probable quenching mechanism between N-CDs and CFX

**Fig. 7:**
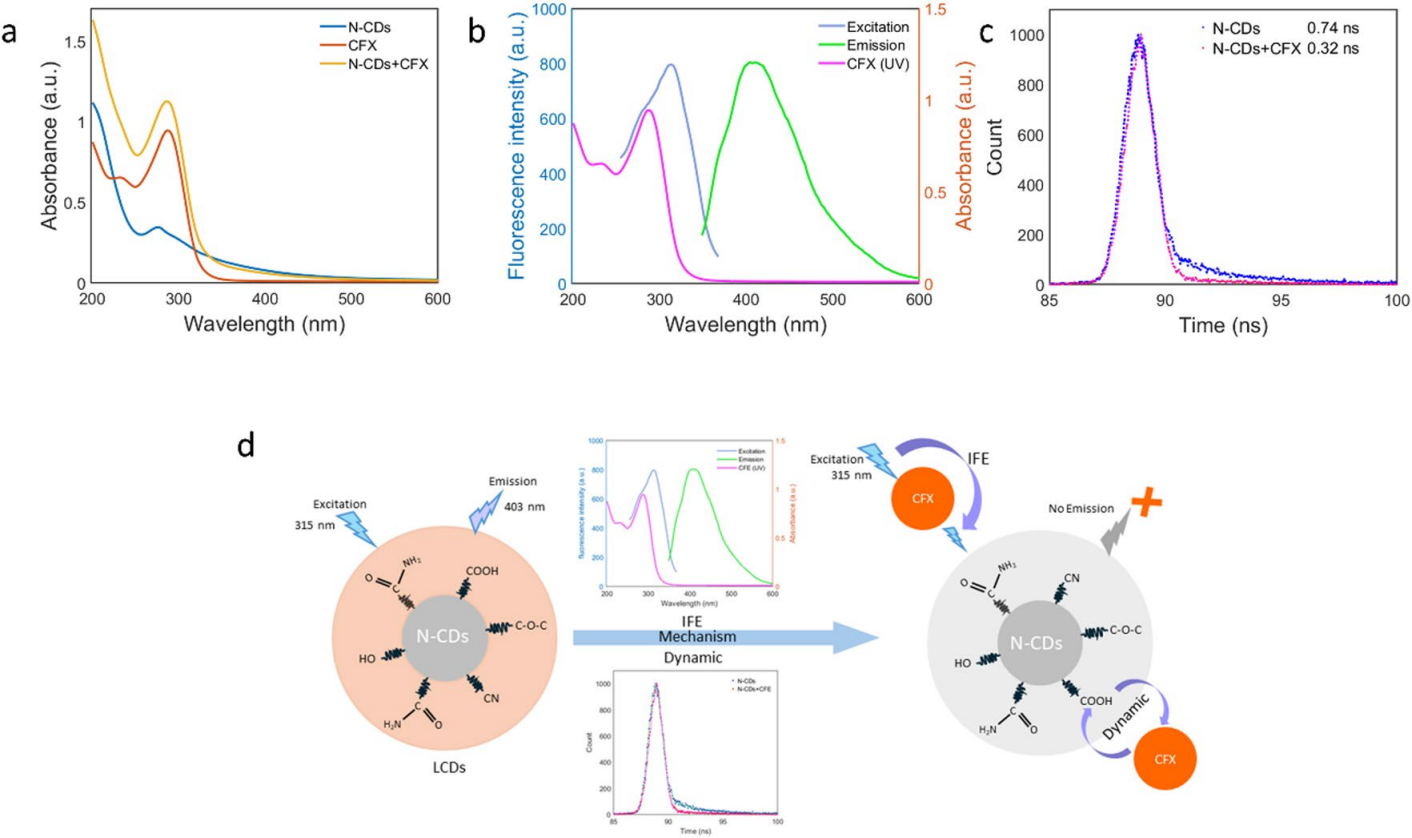
**a** An Overlapping of absorption spectra of CFX with excitation spectra of N-CDs, **b** UV − Vis absorption spectra of N-CDs, CFX, and N-CDs + CFX, (c) Lifetime decay curves of N-CDs and N-CDs + CFX, **d** Schematic of a plausible Sensing mechanism of synergistic combined IFE and dynamic quenching.

Several spectroscopy evaluations were conducted to investigate and predict the quenching mechanism between N-CDs and CFX. As shown in Fig. 7a, the UV–Vis absorption spectra of CFX, N-CDs, and the N-CDs + CFX mixture were analyzed. The resulting spectrum of the mixture appears to be a simple superposition of the individual spectra of CFX and N-CDs, without the emergence of any new absorption peaks. This observation suggests the possibility of both inner filter effect (IFE) and dynamic quenching mechanisms^72^. Further evidence of IFE is provided in Fig. 7b, where a significant overlap is observed between the absorption spectrum of CFX and the excitation spectrum of N-CDs. Such spectral overlap supports the presence of IFE as a contributing mechanism^73^. Fluorescence lifetime measurements were conducted to evaluate whether dynamic quenching is also involved Fig. 7c; Table 3. The average fluorescence lifetime of N-CDs alone was 0.74 ns, which decreased to 0.32 ns in the presence of CFX. The reduction in fluorescence lifetime provides strong evidence for the contribution of a dynamic quenching mechanism^74^. A linear Stern–Volmer relationship was observed in the low OD range of 25 to 1000 nM, resulting in a KSV value of 0.047 µM⁻¹ (R² = 0.995), **Fig. S3**. The intercept, slightly greater than one (~ 1.025), suggests minor inner-filter or background absorption. Additionally, the reduction in lifetime provides evidence of a dynamic component^75,76^. Taken together, the spectroscopic evidence strongly supports a synergistic combined quenching mechanism involving both the inner filter effect (IFE) and dynamic quenching between N-CDs and CFX. Figure 7d shows a schematic of the mechanism.

**Table 3:** Fluorescence lifetime parameters of N-CDs in the presence of CFX.

System	τ₁ (ns)	a₁	τ₂ (ns)	a₂	<τ_av>(ns)	χ²
N-CDs	0.147 ± 0.021	0.786	2.90 ± 0.07	0.214	0.74	1.108
N-CDs + CFX	0.183 ± 0.016	0.953	3.20 ± 0.26	0.047	0.32	1.019

### Detection of CFX in real samples

Tap water and honey were analyzed using two modes (fluorescence and spectrophotometric) and two readouts (paper test strips and a smartphone). For each format, four CFX spike levels were measured using that format’s calibration; Table 4 reports the unspiked sample and two representative spikes within the linear range. Unspiked samples showed no detectable CFX. Across matrices and formats, recoveries were 94.2–108.8% with RSD ≤ 1.9%, confirming accurate and precise quantification.

**Table 4:** Application of the fabricated sensor for CFX detection in real samples (*n* = 3).

Mode	Added (µM)	Tap water		Honey	
		Found (µM)	Recovery ± RSD (%)	Found (µM)	Recovery ± RSD (%)
Spectrophotometric	0	Not Found	-	Not Found	-
	0.5	0.50	100.11 ± 0.62	0.50	99.09 ± 0.64
	1	1.05	105.28 ± 0.53	0.98	98.3 ± 0.31
Fluorescence	0	Not Found	-	Not Found	-
	0.5	0.50	100.45 ± 0.45	0.54	108.83 ± 0.48
	1	1.02	101.99 ± 0.36	0.98	98.48 ± 0.53
Test Strip	0	Not Found	-	Not Found	-
	2.5	2.49	99.69 ± 1.87	2.36	94.21 ± 0.88
	3.5	3.47	99.08 ± 1.04	3.43	97.89 ± 1.58
Smartphone	0	Not Found	-	Not Found	-
	2.5	2.50	100.1 ± 1.22	2.49	99.69 ± 1.33
	3.5	3.49	99.84 ± 0.94	3.47	99.08 ± 1.12

### Selectivity and interference

**Fig. 8:**
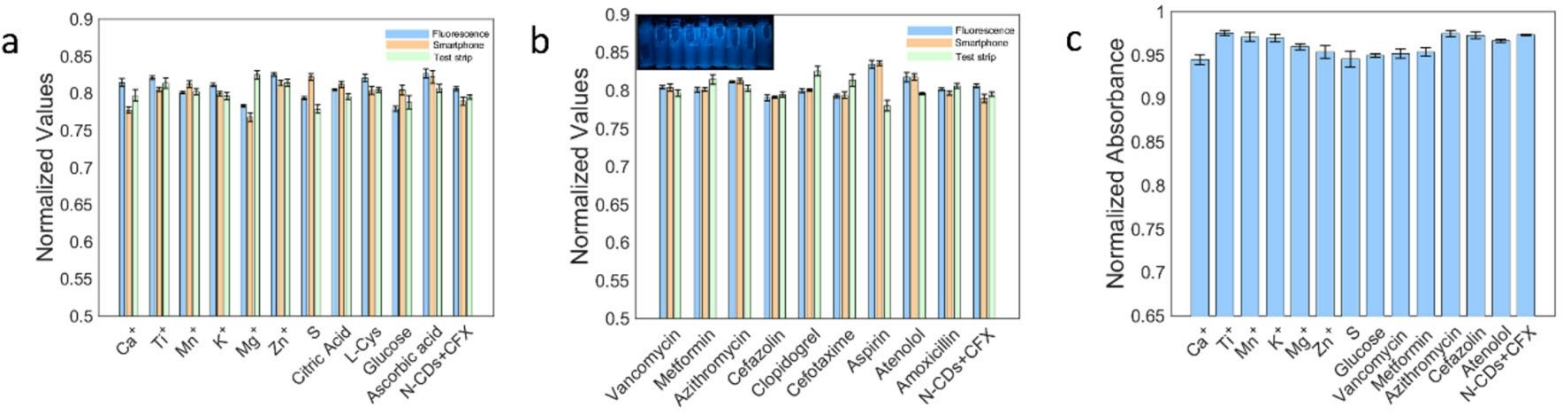
**a** Normalized response in the presence of common metal ions and other potential interferents. **b** Normalized response in the presence of representative drugs; inset: a smartphone photograph of the cuvettes. **c** Selectivity evaluated by the spectrophotometric (normalized absorbance).

Interference studies were performed at a fixed CFX concentration, with each potential interferent added at a 100-fold molar excess (interferent: CFX = 100:1). Under the optimized conditions, responses were recorded in all four formats (two optical mode: fluorescence and spectrophotometric with two readouts: smartphone and paper test-strip), and all signals were normalized to the CFX-only control (= 1.0). As shown in Fig. 8a, common inorganic ions and small molecules (Ca²⁺, Ti⁴⁺, Mn²⁺, K⁺, Mg²⁺, Zn²⁺, S, citric acid, L-cysteine, glucose, ascorbic acid) produced no discernible change relative to the control. Figure 8b shows that co-medications/antibiotics (cefazolin, cefotaxime, amoxicillin, aspirin, atenolol, clopidogrel, vancomycin, metformin, azithromycin) likewise caused no appreciable interference. Consistent trends were observed in the spectrophotometric mode (Fig. 8c). Together, these findings demonstrate the excellent selectivity of the N-CDs platform for CFX determination in complex sample matrices.

## Conclusion

In summary, a novel platform was also developed for CFX detection simultaneously using fluorescence, spectrophotometric, a smartphone, and a test strip as a rapid test. Synthesized N-CDs from onion juice with urea as a dopant had a quantum yield of 72.4%. The fluorescence intensity of N-CDs was dramatically quenched by adding CFX, achieved by combining a dynamic and IFE process, with an LOD of 5.16 nM. Moreover, the developed spectrophotometric mode displayed a wide linear range with a LOD of 138.08 nM. More importantly, the variation in color intensity of the smartphone and test strip readouts in the presence of CFX can be distinguished by processing with the ImageJ software. The LODs were 0.56 µM and 0.65 µM for CFX detection, respectively. Furthermore, the dual-mode fluorescence–spectrophotometric and smartphone and test-strip readouts prob was applied for real samples such as honey, and tap water with satisfactory recoveries. As a result, the designed platform could serve as a convenient and effective approach for accurate detection based on carbon dots.

## Supplementary Information

Below is the link to the electronic supplementary material.

Supplementary Material 1

## Data Availability

The datasets generated and/or analysed during the current study are not publicly available but are available from the corresponding author on reasonable request.
