# Supplementary material for: Dual mode fluorescence and spectrophotometric cefixime sensing using onion juice nitrogen doped carbon dots with smartphone and paper strip readouts
Source: Sci Rep. 2025 Dec 12;15:44626. doi: 10.1038/s41598-025-31426-y (PMC12749416; doi:10.1038/s41598-025-31426-y)
Supplement: Supplementary file 1 — Supplementary Material 1 [file 41598_2025_31426_MOESM1_ESM.docx]

**Supporting Information**

**Title: Dual mode fluorescence and spectrophotometric cefixime sensing using onion juice nitrogen doped carbon dots with smartphone paper strip readouts**

**Authors**

Shahin Asadi*¹ , Negar Ziraksaz*¹

¹ UNAM — National Nanotechnology Research Center and Institute of Materials Science and Nanotechnology, Bilkent University, Ankara,Turkey

Emails:

Shahin Asadi: [shahin.asadi.chem@gmail.com](mailto:shahin.asadi.chem@gmail.com)

Negar Ziraksaz: [negarziraksaz@gmail.com](mailto:negarziraksaz@gmail.com)

**Table S1**. Input design table (Design-Expert v13) used for BBD optimization of N-CDs.

|  |  | Factor 1 | Factor 2 | Factor 3 | Response 1 |
| --- | --- | --- | --- | --- | --- |
| Std | Run | A: microwave (w) | B: time (min) | C: urea (g) | onion-urea |
| 5 | 1 | 500 | 6 | 0.1 | 71.5 |
| 17 | 2 | 700 | 6 | 0.3 | 790.49 |
| 12 | 3 | 700 | 9 | 0.5 | 913.53 |
| 10 | 4 | 700 | 9 | 0.1 | 369.75 |
| 11 | 5 | 700 | 3 | 0.5 | 668.705 |
| 14 | 6 | 700 | 6 | 0.3 | 795.16 |
| 16 | 7 | 700 | 6 | 0.3 | 797.44 |
| 7 | 8 | 500 | 6 | 0.5 | 609.06 |
| 6 | 9 | 900 | 6 | 0.1 | 326.062 |
| 3 | 10 | 500 | 9 | 0.3 | 676.2 |
| 13 | 11 | 700 | 6 | 0.3 | 793.13 |
| 4 | 12 | 900 | 9 | 0.3 | 704.5 |
| 1 | 13 | 500 | 3 | 0.3 | 68.71 |
| 2 | 14 | 900 | 3 | 0.3 | 685.8 |
| 9 | 15 | 700 | 3 | 0.1 | 67.885 |
| 8 | 16 | 900 | 6 | 0.5 | 999.13 |
| 15 | 17 | 700 | 6 | 0.3 | 825.64 |
|  |  |  |  |  |  |

**Table S2**. Results of analysis of variance for the quadratic model in the synthesis process of N-CDs.

| **Source** | **Sum of Squares** | **df** | **Mean Square** | **F-value** | **p-value** |  |
| --- | --- | --- | --- | --- | --- | --- |
| Model | 1.459E+06 | 9 | 1.621E+05 | 525.37 | < 0.0001 | significant |
| A-microwave | 2.080E+05 | 1 | 2.080E+05 | 674.33 | < 0.0001 |  |
| B-time | 1.720E+05 | 1 | 1.720E+05 | 557.42 | < 0.0001 |  |
| C-urea | 6.934E+05 | 1 | 6.934E+05 | 2247.71 | < 0.0001 |  |
| AB | 86668.42 | 1 | 86668.42 | 280.95 | < 0.0001 |  |
| AC | 4590.60 | 1 | 4590.60 | 14.88 | 0.0062 |  |
| BC | 813.39 | 1 | 813.39 | 2.64 | 0.1484 |  |
| A² | 76793.13 | 1 | 76793.13 | 248.94 | < 0.0001 |  |
| B² | 72831.62 | 1 | 72831.62 | 236.09 | < 0.0001 |  |
| C² | 1.131E+05 | 1 | 1.131E+05 | 366.59 | < 0.0001 |  |
| **Residual** | 2159.40 | 7 | 308.49 |  |  |  |
| Lack of Fit | 1335.07 | 3 | 445.02 | 2.16 | 0.2355 | not significant |
| Pure Error | 824.33 | 4 | 206.08 |  |  |  |
| **Cor Total** | 1.461E+06 | 16 |  |  |  |  |

| **Std. Dev.** | 17.56 |  | **R²** | 0.9985 |
| --- | --- | --- | --- | --- |
| **Mean** | 597.81 |  | **Adjusted R²** | 0.9966 |
| **C.V. %** | 2.94 |  | **Predicted R²** | 0.9845 |
|  |  |  | **Adeq Precision** | 69.8754 |


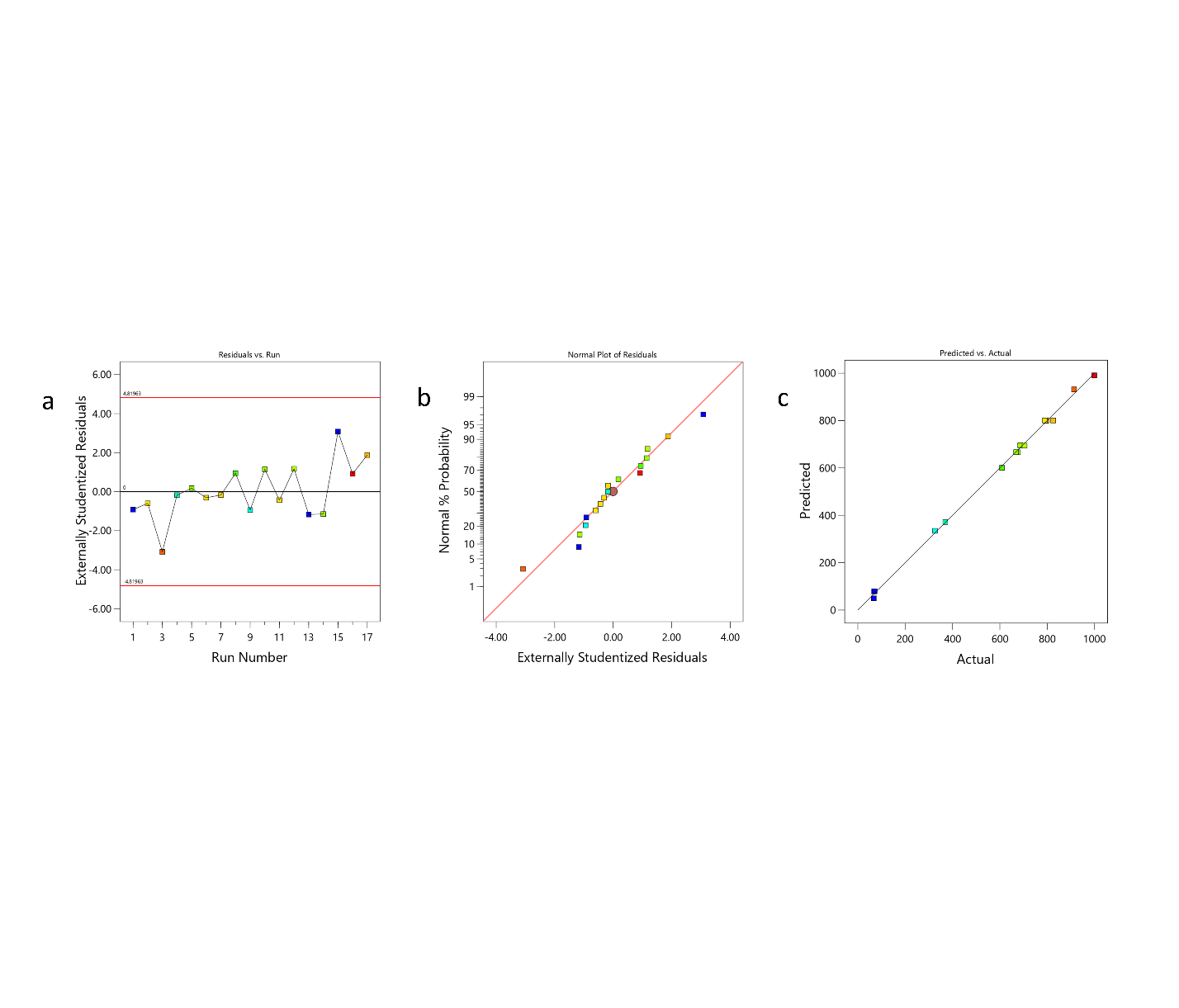


**Fig. S1** a plot of the externally studentized residuals vs. run number. b Normal plot of residuals. c The predicted vs. observed response.


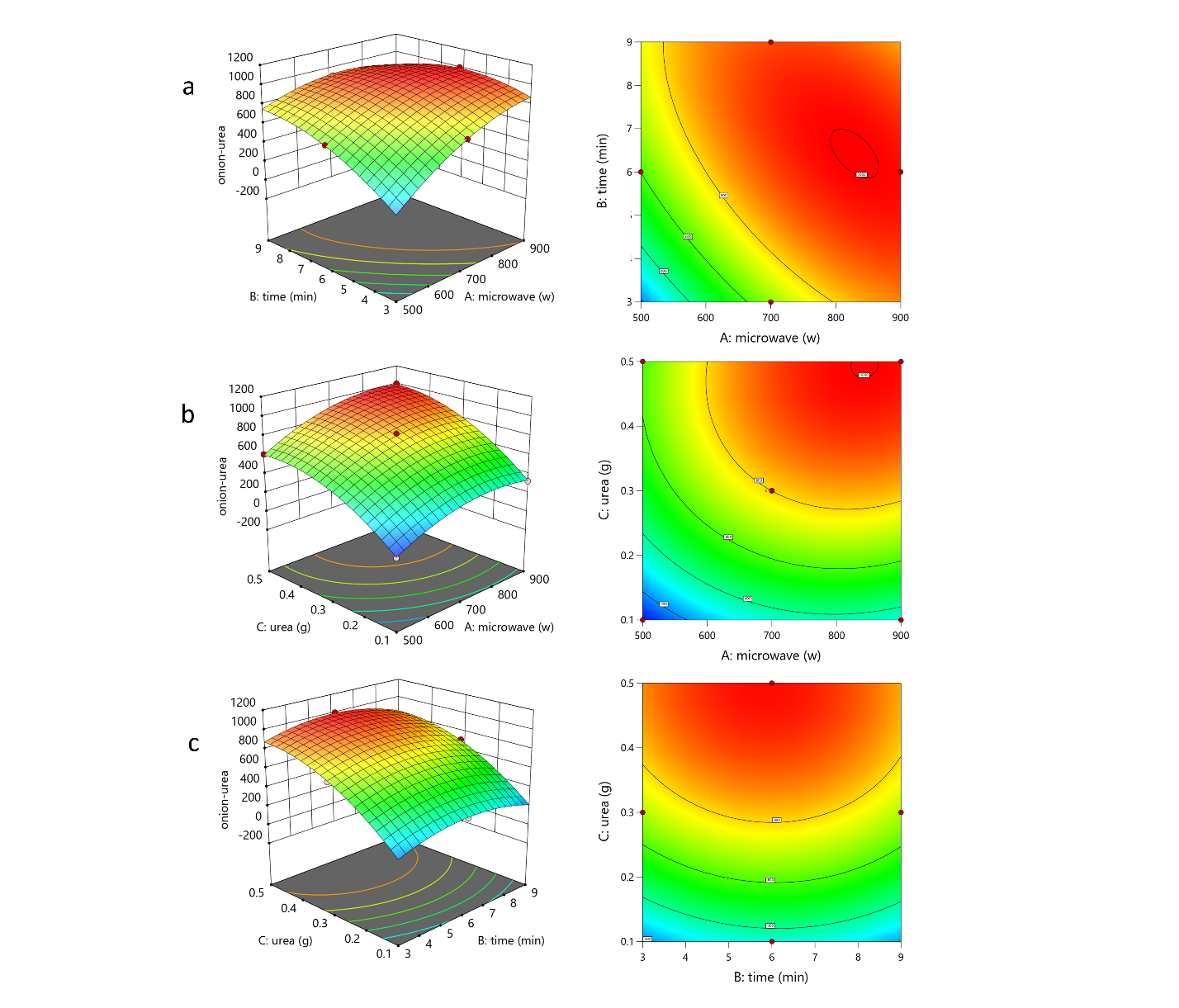


**Fig. S2** Box–Behnken (BBD) 3D response surfaces and contour plots for: (a) irradiation time vs. microwave power; (b) urea amount vs. microwave power; (c) urea amount vs. irradiation time.


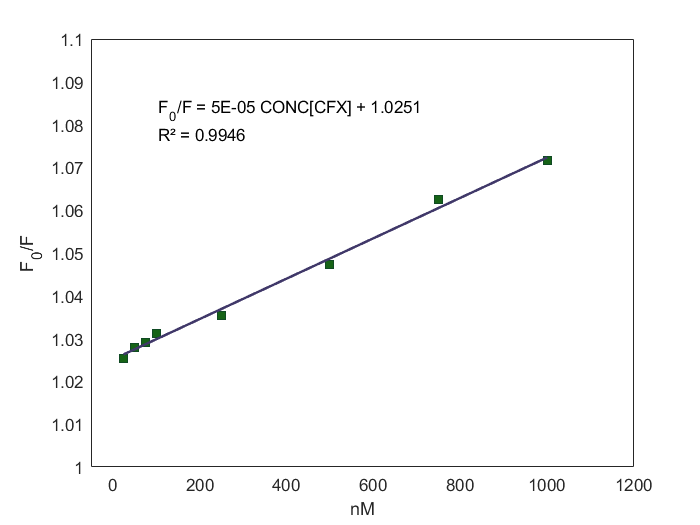


**Fig. S3** Stern–Volmer plot for N-CDs quenched by CFX: F₀/F vs CFX (25–1000 nM), linear fit gives KSV = 0.047 µM⁻¹ (R² = 0.995; intercept ≈ 1.025) under optimized conditions (25 °C, fixed [N-CDs]).
